# The power of a critical heat engine

**DOI:** 10.1038/ncomms11895

**Published:** 2016-06-20

**Authors:** Michele Campisi, Rosario Fazio

**Affiliations:** 1 NEST, Scuola Normale Superiore & Istituto Nanoscienze-CNR, Pisa I-56126, Italy; 2 ICTP, Strada Costiera 11, Trieste 34151, Italy

## Abstract

Since its inception about two centuries ago thermodynamics has sparkled continuous interest and fundamental questions. According to the second law no heat engine can have an efficiency larger than Carnot's efficiency. The latter can be achieved by the Carnot engine, which however ideally operates in infinite time, hence delivers null power. A currently open question is whether the Carnot efficiency can be achieved at finite power. Most of the previous works addressed this question within the Onsager matrix formalism of linear response theory. Here we pursue a different route based on finite-size-scaling theory. We focus on quantum Otto engines and show that when the working substance is at the verge of a second order phase transition diverging energy fluctuations can enable approaching the Carnot point without sacrificing power. The rate of such approach is dictated by the critical indices, thus showing the universal character of our analysis.

## Abstract (web summary)

The second law of thermodynamics says that the efficiency of a heat engine is limited by the Carnot efficiency. Here, the authors use finite-size-scaling theory to investigate whether this ultimate limit can be achieved at finite power using quantum Otto engines.

## Graphical abstract

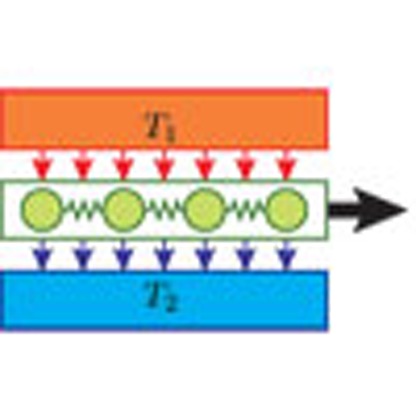

Increasing the power output of a heat engine has a corresponding cost in terms of reduced efficiency *η*, or, in equivalent terms, a larger deviation Δ*η*=*η*^C^–*η* from the Carnot efficiency *η*^C^. A question that is currently the object of vigorous research efforts is whether it is possible to devise a heat engine that outputs finite power at Carnot efficiency^1^,^2^,^3^,^4^,^5^. In the following we address this question by focussing on the quantity


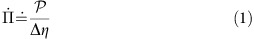


that is, the ratio of power output 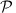 over Δ*η*. We shall call 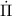 the performance rate. It is trivially possible to increase the power without affecting the efficiency by scaling the size of the working substance (WS): An array of *N* identical engines working in parallel provides an *N*-fold larger power than each of them, at the same efficiency. In this case the output work per cycle and efficiency scale as 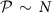, and 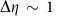, consequently 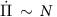 (here the symbol ∼means ‘scales as'). Note that this linear increase in performance rate does not represent any real gain as it is achieved at the cost of a corresponding linear increase of resources. The question is therefore ‘Can the scaling of the performance rate 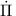 be improved beyond linear' in order to have a true gain? Note that a positive answer implies (in an asymptotic sense) a positive answer to the fundamental question posed above: Imagine having a WS made up of *N* constituents (or resources); if 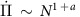, with *a*>0, then one could approach Carnot efficiency as 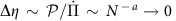 by increasing *N*, while keeping the ‘power per resource' fixed, that is, 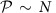.

In the following we make a substantial step towards answering the question above. The main idea that we pursue is that an interaction between the *N* constituents of the WS could provide the extra scaling power that is needed for a positive resolution, see Fig. 1. In order to address that question quantitatively we consider a special engine cycle that is well studied in the literature (see, for example, refs 6, 7, 8 and references therein), namely, a quantum version of the Otto engine^9^,^10^,^11^,^12^,^13^,^14^, see Fig. 2. We show that a universal behaviour, with anomalous scaling of the performance rate


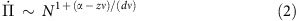


emerges when the WS is on the verge of a second-order phase transition. Here 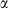,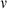,*z* are the specific heat, correlation length and dynamical critical exponent, and *d* is the number of dimensions of the WS. Note that the performance rate contains two concurrent contributions, one stemming from the scaling of the heat capacity, that is, the exponent 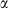, and the other stemming from the scaling of the relaxation time, that is, the exponent *z*. Since *d*,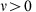, in order to have a more than linear behaviour of 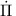 one needs a WS with a critical point characterized by the inequality 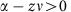. In fact, as we explain below in detail, the stronger condition


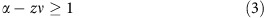


would ensure the asymptotic approach towards the Carnot point without giving up power per resource. Is that possible? The finiteness of internal energy implies the bound 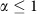. Critical slowing down (*z*≥0) would then imply 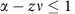 (for example, in the three-dimensional Ising model, 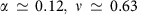 (ref. 15) and 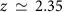 (ref. 16); hence 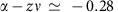). A number of theoretical and experimental works report on the possibility of the exotic phenomenon of critical speeding up, *z*≤0 (refs 17, 18, 19, 20). In this case the two terms might add up above unity (for example, recent experimental studies report 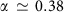 (ref. 21) and 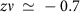 (ref. 19); hence 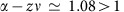, for Dy_2_Ti_2_O_7_). We conclude that there currently appears to be no fundamental reason hindering the possibility of approaching Carnot efficiency without giving up power per resource. This is certainly possible up to some threshold size 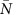 for which the weaker condition 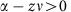 suffices. Below we illustrate how such powerful critical engines should be designed. At the heart of our result is the recognition that scaling of the performance, that is,


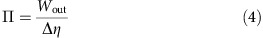


where *W*_out_ is the work output per cycle, is dictated by the heat capacity of the WS (which can notably diverge at the critical point), a crucial and simple fact that was never noticed before, see equation (9).

## Results

### N-body quantum Otto engine

**Figure 2:**
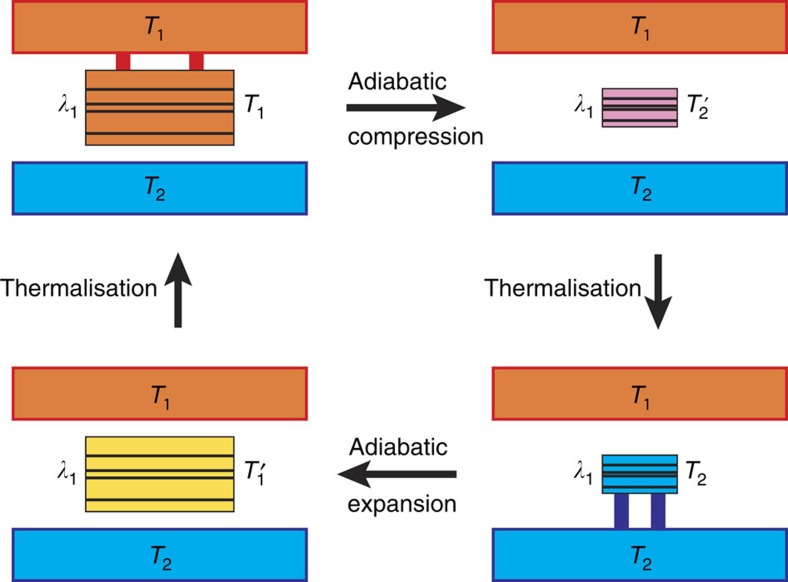
A quantum Otto engine. At time *t*=0 the WS is in thermal equilibrium with bath 1 and *λ*=*λ*_1_. During the first stroke the WS undergoes a thermally isolated transformation where the Hamiltonian switches from 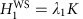 to 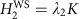. The second stroke consists in thermalization with bath 2, with *λ* being kept fixed at *λ*_2_. During the third stroke the Hamiltonian goes back to 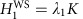 while in thermal isolation. The fourth stroke consists in letting the system thermalize with bath 1, with *λ* being kept fixed at *λ*_1_.

The quantum Otto engine (see Fig. 2) is a four-stroke engine based on a WS with Hamiltonian


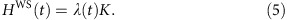


(Following the current literature we call these engines ‘quantum Otto engines' although they bear no other quantum feature besides the discreteness of the spectrum. Accordingly, there is nothing genuinely quantum in our treatment.) The heats released in the baths during the thermalization strokes are 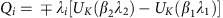, where 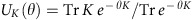 is the internal energy associated to the base Hamiltonian *K* at inverse temperature *θ*, and −, + is for *i*=1, 2, respectively. The work performed by the engine *W*_out_=*Q*_1_+*Q*_2_ is thus


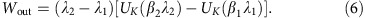


For 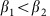, the condition *W*_out_≥0, *Q*_1_≥0 defines the regime of operation of the engine as a heat engine, implying 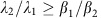. The efficiency is


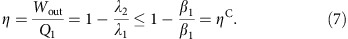


The efficiency is smaller than *η*^C^, in accordance with the heat engine fluctuation relation^22^,^23^.

Using equation (7) the output work of the quantum Otto engine can be expressed as a function of 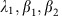 and 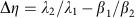 as


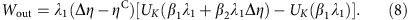


**Figure 3:**
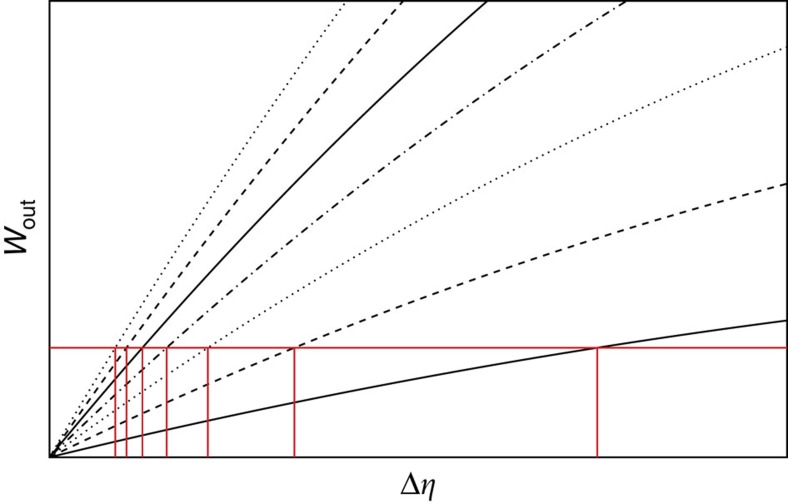
Graphical demonstration of equation (9). The graph shows various plots of *W*_out_(Δ*η*) for increasing values of *N* (from lower to upper curves). At a fixed work output (horizontal red line), the curves are intercepted at decreasing values of Δ*η*. If the slope at the origin increases with a power *N*^*a*^, 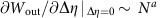; then, provided for larger and larger *N* the intercept occurs in the region where the linear approximation holds for the *N*th curve, the approach towards Δ*η*=0 occurs as 1/*N*^*a*^; hence 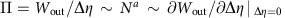. Similarly, one might increase (lower) the value of *W*_out_ with *N*, for example, as *W*_out_(Δ*η*)=*wN*^*b*^. Accordingly, provided for larger and larger *N* the intercept still occurs in the region where the linear approximation holds, the approach towards Δ*η*=0 occurs as 1/*N*^*a*–*b*^. Still, 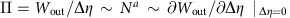.

This expression allows us to study the performance Π. In the region where linear approximation holds, that is, 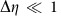 (namely 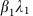 is close to 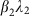), the scaling of the engine's performance Π with *N* is given by the scaling of ∂*W*_out_/∂Δ*η*|_Δ*η*=0_, that is, the slope of *W*_out_(Δ*η*) at the origin. This is illustrated in Fig. 3. In the case of *N* devices in parallel it is 
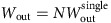
 (here 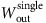 denotes the work of each single device); hence trivially 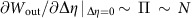, and, accordingly 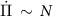, as we noted before. It follows that in order to boost the scaling of the performance it is necessary to have the slope ∂*W*_out_/∂Δ*η*|_Δ*η*=0_ to scale more than linearly.

By taking the derivative of equation (8) with respect to Δ*η* we obtain the central relation


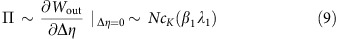


where *c*_*K*_(*θ*)=−(1/*N*)*θ*^2^∂*U*_*K*_/∂*θ* is the specific heat of the WS. To understand the physics behind the emergence of equation (9), consider working at some point that is very close to the Carnot point, that is, chose 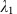 and 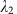 so that their ratio 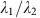 is very close to 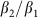. After the adiabatic compression stroke the WS reaches a new temperature 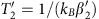, which is very close to the cold bath temperature 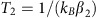: 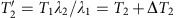, with a small Δ*T*_2_. The larger the heat capacity *C*_*K*_=*Nc*_*K*_ of the WS, at the thermalization point, the larger the heat exchanged *Q*_2_=*C*_*K*_Δ*T*_2_ during the subsequent thermalization with the cold bath. Likewise for the subsequent expansion and thermalization. Since *W*_out_=*Q*_1_+*Q*_2_, the larger the heat capacity the larger the work output, and accordingly the larger the performance.

Equation (9) tells us that in order to achieve super-linear scaling of the performance, one needs a WS with an anomalous scaling of the specific heat. Recall that for ordinary substances the heat capacity is extensive, 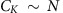, namely 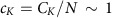. What is needed for improved performance is 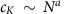, with some *a*>0. This can happen at the verge of a phase transition. The physical reason is that at a phase transition finite exchanges of heat are accompanied by infinitesimal changes of temperature, that is, the specific heat diverges. This is because at the transition point the energy intake is not employed to heat up but rather to make the change of phase.

For a second-order phase transition, finite size scaling predicts a peak in the specific heat whose height and width scale, respectively, as 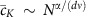 and 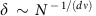 (ref. 24) where *d* is the dimensionality of the system, and *α* and *ν* are the specific heat and the correlation length critical exponents. Accordingly we predict the possibility of a boosted scaling of the perfmormance 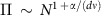. Writing the performance rate as 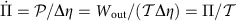 we see that its scaling is determined by the scaling of Π and of the cycle time 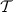. The latter is dominated by the thermalization time^2^, which, according to finite-size scaling theory scales with the dynamical critical exponent *z* as 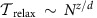 (ref. 25). This gives equation (2).

### Critical engine design

We illustrate how a quantum Otto engine can be designed to achieve the predicted performance rate in equation (2). Let the substance be described by a Hamiltonian *K*, displaying, in the infinite size limit, a second-order phase transition at the inverse critical temperature *θ*_C_. Let the two baths have the inverse temperatures *β*_1_ and *β*_2_. We assume we can stretch/compress the spectrum of the WS and implement accordingly the time-dependent Hamiltonian in equation (5) with *λ*(*t*)∈[*λ*_1_, *λ*_2_]. When *λ* takes on the value *λ*_*i*_, the critical temperature is rescaled to *β*_C_^*i*^=*θ*_C_/*λ*_*i*_. We choose, for example, *λ*_1_ so that 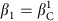 (that is 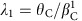). In this way the temperature of bath 1 coincides with the critical temperature of 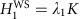. We next choose *λ*_2_ as the solution of *W*_out_=*N*^1+*z*/*d*^*w*, for some fixed *w*. That gives the corresponding efficiency *η*=1–*λ*_2_/*λ*_1_. Note that since 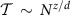, the power per constituent is fixed: 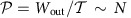. Since the slope of the graph *W*(Δ*η*) grows with the heat capacity as 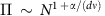, we have 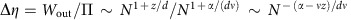. Hence if *α*–*νz*>0 the solution *λ*_2_ gets closer and closer to *λ*_1_*β*_1_/*β*_2_. Accordingly the efficiency *η*=1–*λ*_2_/*λ*_1_ gets closer and closer to *η*^C^=1–*β*_1_/*β*_2_. As discussed in the caption of Fig. 3, the previous statement is correct as long as *λ*_2_ falls in the region of validity of the linear approximation (that is, in graphical terms, if the corresponding Δ*η* falls in the region where the curve *W*(Δ*η*) is well approximated by a straight line passing through the origin). The extension of that region corresponds to the width of the specific heat peak, which, as mentioned above, universally scales as 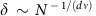. So, in order to keep pace with the shrinking of the linear approximation region, the approach towards Δ*η*=0 should be not slower than *N*^−1/(*dν*)^. Since that would occur at a pace of *N*^*−*(*α*–*νz*)/(*dν*)^, the condition ensuring that it actually asymptotically occurs is given by equation (3).

**Figure 4:**
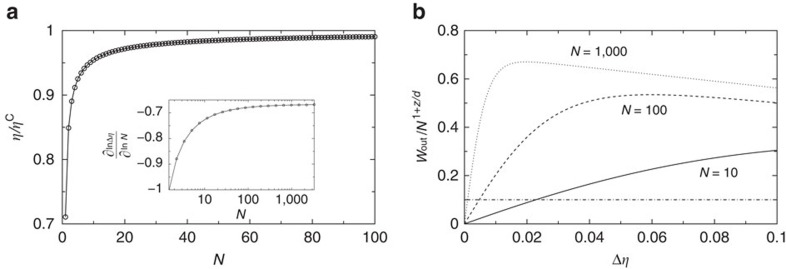
Efficiency and shrinking of linear region in a critical sped-up engine. (**a**) Approach towards the Carnot point of a critical working substance characterized by equation (3) at a fixed power per constituent. Plots were calculated with critical indices reported recently for Dy_2_Ti_2_O_7_, namely 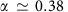 (ref. 21) and 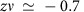 (ref. 19). The value of *ν* was obtained from the relation *ν*=(2–*α*)/*d* (ref. 27). Carnot point was at *η*^C^=1/2 and *w*=*W*_out_/*N*^1+*z*/*d*^=0.1. The inset shows the quantity ∂ ln Δ*η*/∂ ln *N* as a function of *N*, that is, the exponent characterizing the approach to the Carnot point that reaches the expected value 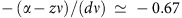. (**b**) Rescaled output work *W*_out_/*N*^1+*z*/*d*^ as a function of Δ*η*. Note that the linear region shrinks around the origin of axes as *N* increases, and that the intercept with *W*_out_/*N*^1+*z*/*d*^=0.1 occurs within that region for all curves.

In Fig. 4a we illustrate how in such a case the engine design that we have described above actually results in an asymptotic approach towards the Carnot point, at a fixed power per constituent. Figure 4b illustrates the shrinking of the linear region, due to the narrowing of the peak width. In making Fig. 4 we have taken full advantage of one of the most striking aspects of our analysis, namely its universality. The details of the specific model are not essential, all that counts are the critical exponents. The specific heat peak reads *c*_*K*_(*θ*)=*N*^*α*/(*dν*)^*θ*^2^*U*_1_Δ*Uf*(Δ*U*(*θ*–*θ*_C_)*N*^1/(*dν*)^), where *f*(*x*) is some bell-shaped function (its exact shape is not relevant). The corresponding internal energy of the WS around the critical point reads *U*_*K*_(*θ*)=*U*_0_–*N*^1+(*α*–1)/(*dν*)^*U*_1_*F*(Δ*U*(*θ*–*θ*_C_)*N*^1/(*dν*)^), where *F*′(*x*)=*f*(*x*). The plots are obtained by using the latter formula, with equation (6) used to evaluate the work output, with the choice *f*(*x*)=sech^2^(*x*), *F*(*x*)=tanh(*x*) and *U*_1_=Δ*U*_1_=1. The plot illustrates the approach towards Carnot efficiency at a fixed power per constituent, with the predicted scaling exponent −(*α*–*zν*)/(*dν*).

## Discussion

The idea that phase transitions could enable the attainment of Carnot efficiency at finite power was previously hinted by Polettini *et al.*^5^, but was never pursued before. The present work confirms that intuition in a fully fledged way based on universality and finite-size scaling theory and most importantly by accounting for the first time for the effect of criticality on the time of operation hence of the power.

As is clear from Fig. 3, independent of how the slope ∂*W*_out_/∂Δ*η*|_Δ*η*=0_ scales, *W*_out_(Δ*η*=0)=0; hence, exactly at the Carnot point all quantum Otto engines deliver null work and hence null power. Our statement should be accordingly understood in a weaker asymptotic sense, namely, that it is possible to get as close as one wants to the Carnot point without giving up the power per constituent. This is the viewpoint that also inspires ref. 2.

We remark that our linear condition 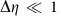 substantially differs from the condition of linear response regime (that is, 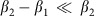 or 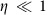) that was investigated previously in refs 1, 3, 4, 5. In our case the two temperatures need not be close to each other.

We stress that all obstacles hindering the realization of the critical powerful Carnot engines appear to be of technological nature, rather than fundamental. One major difficulty stems from the necessity of implementing the Hamiltonian (5) containing a global coupling *λ*(*t*), which can be very challenging in practice. Another difficulty stems from the fact that in order to be able to asymptotically approach the Carnot point, one should accordingly have an increasing degree of accuracy with which *λ*_1_ and *λ*_2_ are controlled.

Lastly, it is worth stressing that the result in equation (9) holds in general as a consequence of the linear approximation valid for 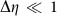, and is accordingly not restricted to the case of critical phenomena. This tells that one route towards the improvement of the performance of a WS is to increase its specific heat. That could be achieved by increasing its number of constituents, a possibility that we have investigated here, or by manipulating any other parameter entering the Hamiltonian *K*. Recently reported cases of improved performances^26^ can in fact be interpreted in terms of increased heat capacity.

### Data availability

The authors declare that all data supporting the findings of this study are available within the article.

## Additional information

**How to cite this article:** Campisi, M. and Fazio, R. The power of a critical heat engine. *Nat. Commun.* 7:11895 doi: 10.1038/ncomms11895 (2016).

## Figures and Tables

**Figure 1:**
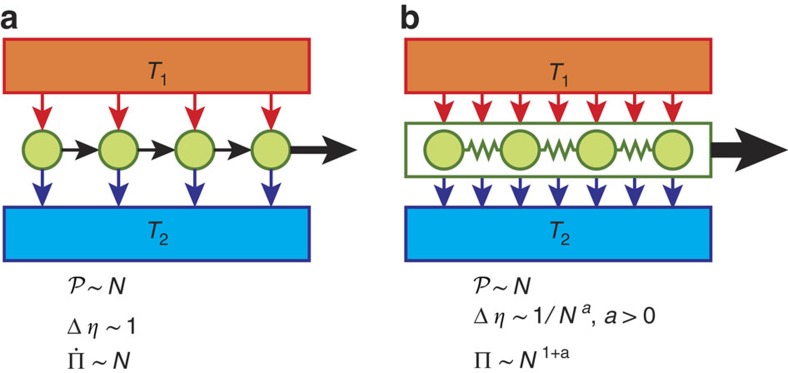
Main idea at the basis of the results. (**a**) *N* identical devices operating in parallel provide a power 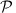 that scales like *N*, at fixed efficiency. (**b**) When an interaction among the *N* parallel devices is turned on, the approach to Carnot efficiency is enabled.
